# The Impact of the Geometric Correction Scheme on MEG Functional Topology at Rest

**DOI:** 10.3389/fnins.2019.01114

**Published:** 2019-10-25

**Authors:** Stefania Della Penna, Maurizio Corbetta, Vincent Wens, Francesco de Pasquale

**Affiliations:** ^1^Department of Neuroscience, Imaging and Clinical Sciences, and Institute for Advanced Biomedical Technologies, “G. d’Annunzio” University of Chieti-Pescara, Chieti, Italy; ^2^Department of Neuroscience and Padova Neuroscience Center, University of Padua, Padua, Italy; ^3^Department of Neurology, Radiology, and Anatomy and Neurobiology, Washington University, St. Louis, MO, United States; ^4^Laboratoire de Cartographie Fonctionnelle du Cerveau, UNI—ULB Neuroscience Institute, Université Libre de Bruxelles, Brussels, Belgium; ^5^Magnetoencephalography Unit, Department of Functional Neuroimaging, Service of Nuclear Medicine, CUB—Hôpital Erasme, Brussels, Belgium; ^6^Faculty of Veterinary Medicine, University of Teramo, Teramo, Italy

**Keywords:** functional connectivity, band-limited power correlation, MEG connectome, leakage correction, functional hubs

## Abstract

Spontaneous activity is correlated across brain regions in large scale networks (RSN) closely resembling those recruited during several behavioral tasks and characterized by functional specialization and dynamic integration. Specifically, MEG studies revealed a set of central regions (dynamic core) possibly facilitating communication among differently specialized brain systems. However, source projected MEG signals, due to the fundamentally ill-posed inverse problem, are affected by spatial leakage, leading to the estimation of spurious, blurred connections that may affect the topological properties of brain networks and their integration. To reduce leakage effects, several correction schemes have been proposed including the Geometric Correction Scheme (GCS) whose theory, simulations and empirical results on topography of a few RSNs were already presented. However, its impact on the estimation of fundamental graph measures used to describe the architecture of interactions among brain regions has not been investigated yet. Here, we estimated dense, MEG band-limited power connectomes in theta, alpha, beta, and gamma bands from 13 healthy subjects (all young adults). We compared the connectivity and topology of MEG uncorrected and GCS-corrected connectomes. The use of GCS considerably reorganized the topology of connectivity, reducing the local, within-hemisphere interactions mainly in the beta and gamma bands and increasing across-hemisphere interactions mainly in the alpha and beta bands. Moreover, the number of hubs decreased in the alpha and beta bands, but the centrality of some fundamental regions such as the Posterior Cingulate Cortex (PCC), Supplementary Motor Area (SMA) and Middle Prefrontal Cortex (MPFC) remained strong in all bands, associated to an increase of the Global Efficiency and a decrease of Modularity. As a comparison, we applied orthogonalization on connectomes and ran the same topological analyses. The correlation values were considerably reduced, and orthogonalization mainly decreased local within-hemisphere interactions in all bands, similarly to GCS. Notably, the centrality of the PCC, SMA and MPFC was preserved in all bands, as for GCS, together with other hubs in the posterior parietal regions. Overall, leakage correction removes spurious local connections, but confirms the role of dynamic hub regions, specifically the anterior and posterior cingulate, in integrating information in the brain at rest.

## Introduction

The spontaneous activity of the brain at rest is spatially and temporally organized in large-scale networks of cortical and subcortical regions, denoted Resting State Networks (RSNs). The topography of RSNs is similar to that of brain networks recruited by different cognitive tasks (Biswal et al., 1995; Attwell and Laughlin, 2001; Fox et al., 2005), and for this reason RSNs have been named according to their putative function and pattern of task activation: dorsal and ventral attention, visual, somato-motor, auditory, language, executive control, and default systems (Doucet et al., 2011; Hacker et al., 2013; Glasser et al., 2016).

As behavior unfolds, these functionally specific systems must integrate to ensure efficient processing and transfer of information in the brain. This seems to be achieved through a specialized architecture of brain regions, as shown by fMRI, DTI, EEG and MEG studies (van den Heuvel and Sporns, 2013; de Pasquale et al., 2018). A possible mechanism allowing for the dynamic integration of activity in different brain regions is the existence of structural and functional ‘hub’ regions that, through the architecture of their interactions, structural, as shown by DTI studies (van den Heuvel et al., 2012), and functional, as shown by fMRI studies (Zuo et al., 2012; Power et al., 2013), act as waystations of integration thus facilitating the communication within/across RSNs. Studies at higher temporal resolution, using for instance MEG, revealed a more complex scenario where nodes of the Default Mode, Dorsal Attention and Somato-Motor Networks act as dynamic cortical cores of integration in the beta and alpha bands (de Pasquale et al., 2010, 2012, 2018). Furthermore, these areas are not independent but their centrality tend to co-fluctuate, hence forming a so called “dynamic core network of interaction” (de Pasquale et al., 2016). These findings nicely link with other MEG studies, showing a temporally varying organization of brain subnetworks – MEG states- (Baker et al., 2014), and with the DTI-supported notion of a structural Rich Club organization within the brain connectome. Many of these functional and structural models strongly depend on which measures of connectivity are adopted, e.g., correlation-based measures of interaction, and on which measures are used to analyze graph properties. In the case of MEG, even though neuromagnetic signals have broader frequency content and higher temporal resolution than fMRI, and are not influenced by neurovascular coupling, a serious drawback is the inherent “spatial leakage” which generates a spurious codependence among the reconstructed activity of distinct sources. In fact, in order to solve the ill-posed inverse problem, linear source projection schemes such as MNE (Hamalainen and Ilmoniemi, 1994) or Beamformers (Van Veen et al., 1997; Hillebrand et al., 2005) will inevitably yield a spatially blurred representation of the underlying source distribution, with signals reconstructed at different locations affected by activity from neighboring brain areas. These effects will largely affect the connectivity estimation. To overcome this problem, several measures insensitive to spatial leakage have been introduced: the imaginary coherence (Nolte et al., 2004), the multivariate interaction measure (Marzetti et al., 2013) or the phase lag index (Stam and Reijneveld, 2007; Hillebrand et al., 2012) for phase coupling on the fast signal (activity), the orthogonalized correlation (Brookes et al., 2012; Hipp et al., 2012; O’Neill et al., 2015) and the symmetrical multivariate leakage corrections (Colclough et al., 2015) for amplitude coupling on the slow signal (activity envelope). The common idea behind all these correction schemes is that spatial leakage can only induce zero-lag linear spurious coupling, which can be in turn eliminated by an appropriate regression model (Wens, 2015), and that it does not affect non-zero-lag connectivity (see Palva et al., 2018 for a critical overview on this assumption). However, a potential issue with these approaches is that physiological interactions involving zero-lag linear coupling may be suppressed as well. This is particularly important since, with synaptic delays in the range of 5–25 ms from neighboring to remote regions, zero-lag interactions are expected to be physiologically dominant. This has been widely documented in empirical data as well as modeling studies (Gollo et al., 2014). In fact, these mechanisms have been ascribed to a range of crucial neuronal functions, from perceptual integration to the execution of coordinated motor behaviors (Roelfsema et al., 1997; Singer, 1999; Varela et al., 2001; Uhlhaas et al., 2009). A recent work also suggests the existence of zero-lag correlations at rest, specifically within the Default Mode Network (Sjogard et al., 2019).

A possible alternative method for preserving zero-phase lag correlation is the Geometric Correction Scheme (GCS) proposed by Wens et al. (2015), which models spatial leakage from a seed location based on the forward and inverse models. The fundamental theoretical aspects of the GCS as well as simulation- and data-based proof-of-concept were developed in Wens (2015) and Wens et al. (2015), but they were limited to the study of RSN topographies. Here, we investigate the effect of this leakage correction on a dense connectome (155 nodes, involving 9 separate RSNs) as a function of the oscillatory band and the eventual impact on the estimated topological features. Specifically, we estimated the dense connectome based on band-limited power (BLP) computed in the theta, alpha, beta and gamma bands without leakage correction and with the GCS. We first compared the overall topology through Network Based Statistics (NBS). Furthermore, we considered topological features such as the Betweenness Centrality and Global Efficiency, to understand the impact of the GCS on the identification of hub regions and the efficiency of integration. Our hypothesis is that GCS will affect local connections, producing a decrease of local links, and a partial reorganization of the brain hubs. We expect that leakage reorganization will occur mainly in the alpha and beta bands, which are the main correlates of fMRI RSNs (Mantini et al., 2008). Finally, to assess the impact on the topology of removing 0-lag correlations as compared to maintaining them, we ran the same analyses also on connectomes where leakage was corrected through orthogonalization similarly to Brookes et al. (2012). Based on the overall similarity of RSN topography shown in Wens et al. (2015), we expect to find some agreement between results obtained from the two approaches.

## Materials and Methods

### Subjects and Recordings

MEG signals were recorded from 13 healthy adult subjects (mean age 29 ± 6 years, 5 females), already used in de Pasquale et al. (2016). Subjects were asked to remain still in the MEG system while fixating a cross on a screen. We recorded 2 or 3 scans lasting 5 min from each subject. MEG signals were recorded using the 153-channel MEG system developed and installed at the University of Chieti (Della Penna et al., 2000). The system is placed within a four-layer magnetically shielded room allowing magnetometric recordings. Two EOG and two ECG channels were recorded simultaneously with the MEG signals, to be used for physiological artifact rejection. Neuro-magnetic and electrical signals were filtered in the band 0.16–250 Hz and were sampled at 1 kHz. Before and after each resting state run, the signal generated by five positioning coils placed on the subject’s head were recorded and used to co-register functional and anatomical data. Anatomical images were acquired using a 1.5 T Siemens Vision scanner, through a sagittal magnetization-prepared rapid acquisition gradient echo T1-weighted sequence (MP-RAGE) with repetition time (TR) = 9.7 s; echo time (TE) = 4 ms; α = 12°; inversion time = 1,200 ms; voxel size = 1 × 1 × 1.25 mm^3^.

### MEG Data Preprocessing

An extensive description of the approach used to preprocess our MEG data can be found in Mantini et al. (2011). In brief, we applied a pipeline based on ICA (similar to the one described in Larson-Prior et al., 2013) to separate and identify environmental and physiological (e.g., cardiac, ocular) artifacts, which were removed from sensor-space MEG data. Runs affected by excessive noise (e.g., movement of the subject’s head in the helmet) were discarded from further analysis. This reduced our final sample to a total of 27 runs. The sensor maps of non-artifactual ICs (brain ICs) were projected into the individual source space through a weighted minimum norm estimator using Curry 6 (Neuroscan, Hamburg, Germany). The source space was mapped into a 3D grid of cubic voxels with a 4 mm side, coregistered to the MNI atlas. The vector activity $\text{Ψ}\,\left(t\right)=\sum_{\text{ic}}{\text{A}}_{\text{ic}}\,s_{\text{ic}}\,\left(t\right)$ at each voxel in the brain was obtained as the linear combination of the brain IC timecourses weighted by the related source-projected IC maps.

### Connectivity Estimation and Geometric Leakage Correction

We estimated a dense connectome based on BLP correlations among a set of 155 nodes obtained in a previous meta-analysis on fMRI data (Hacker et al., 2013; Baldassarre et al., 2014), which yielded 9 RSNs (Dorsal Attention Network –DAN, Ventral Attention Network – VAN, Somato-Motor Network – SMN, Visual Network – VIS, Auditory Network – AUD, Language Network – LAN, Default Mode Network – DMN, Fronto-Parietal Network – FPN, Control Network – CON). Subcortical ROIs were removed from the original set. Source activity ***Ψ***(*t*) was filtered into 4 frequency bands: theta (4–7 Hz), alpha (7–14 Hz), beta (14–25 Hz), gamma (25-70 Hz) and their BLP time series were obtained by integrating the square amplitude of the signal over 150 ms windows sliding by 20 ms. Then, for each pair of nodes, we computed the Pearson correlation coefficient between their respective BLP time series over non-overlapping epochs lasting 40 s and then averaged them across the whole run. This procedure yielded the original, uncorrected connectivity matrices. To obtain their leakage-corrected version, each node was successively taken as a seed and spatial leakage emanating from it was modeled using the GCS (Wens, 2015; Wens et al., 2015), which we review in the following.

The GCS was applied on the source space maps of each brain IC before estimating the leakage-corrected activity as follows. Given a seed **r**_0_, and the linear inverse operator $\text{W}=\sum_{i\,c}{\text{A}}_{i\,c}\,{\text{u}}_{i\,c}$ associated with our source reconstruction pipeline (Mantini et al., 2011; Betti et al., 2018), where **A**_*ic*_ is the source-space map for a generic component IC with dimensions (N_voxels_ x 3), coregistered to the MNI atlas, **u**_*ic*_ is the row of the unmixing matrix with dimensions (1 x N_channels_), and the sum is over the brain ICs, the corrected source map was computed as:

$${\text{A}}_{i\,c}^{G\,C\,S}={\text{A}}_{i\,c}-\left(\sum_{i\,c}{\text{A}}_{i\,c}\,{\text{u}}_{i\,c}\,\text{L}\,\left({\text{r}}_{0}\right)\right)\,{\left(\sum_{i\,c}{\text{A}}_{i\,c}\,\left({\text{r}}_{0}\right)\,{\text{u}}_{i\,c}\,\text{L}\,\left({\text{r}}_{0}\right)\right)}^{+}\,{\text{A}}_{i\,c}\,\left({\text{r}}_{0}\right),$$

where **L**(**r**_0_) is the leadfield associated to the seed, with dimensions (N_channels_ x 3) also coregistered to the MNI atlas, and + denotes pseudoinversion. Given the seed, the leakage-corrected vector activity of all the other nodes was then rebuilt as:

$${\text{Ψ}}^{G\,C\,S}\,\left(t\right)=\sum_{\text{ic}}{\text{A}}_{i\,c}^{G\,C\,S}\,s_{i\,c}\,\left(t\right)$$

where *s*_ic_ (*t*) are the brain IC timecourses. The row of the connectivity matrix corresponding to this seed was obtained by correlation of the BLP obtained from the uncorrected activity $\text{Ψ}\,\left(t\right)=\sum_{\text{ic}}{\text{A}}_{\text{ic}}\,s_{\text{ic}}\,\left(t\right)$at the seed and the BLP of all the corrected activities ***Ψ***^*GCS*^(*t*) at the other nodes.

GCS connectomes were then symmetrized by averaging the upper and lower triangles to avoid the slight asymmetries induced by leakage correction. Of note, spatial leakage is symmetrical in minimum norm estimates (Hauk and Stenroos, 2014), so this step discarded only slight asymmetries.

Finally, as suggested in Wens et al. (2015), to remove the contribution of possible local under-correction effects due to seed mis-localization in the GCS connectomes, we masked out node pairs closer than 35 mm. To avoid biasing comparisons between connectomes, this distance mask was applied to both uncorrected and the corrected connectomes. Thus, all the correlation values between node pairs closer than 35 mm were not considered in all the analyses applied to the two types of connectomes and described in the following subsections. The number of node pairs masked in our analysis was 1155, corresponding to less than 5% of the total number of node pairs (equal to 23870).

### Analysis of Topological Effects of Leakage Correction

First, we analyzed possible decreases of correlation strength by estimating the z-Fisher transformed correlation averaged over the whole connectome, for each subject and band, and performing paired *t* -tests assessing the effect of the GCS over each frequency band. Then, topological changes induced by the GCS were investigated through the NBS Toolbox (Zalesky et al., 2010) at each frequency band. NBS directly operates on the connectivity values and searches for graph components (subgraphs made of connected nodes) comprising suprathreshold links obtained from a t-statistics testing connectivity differences. We looked for components representing decreases and increases separately, using a large primary threshold for the t-statistic, namely 6. This value represents a good compromise between the size of the significant components produced by NBS, which was already large for all bands at this threshold, and the sake of clarity in showing and interpreting the obtained components. However, it must be noted that the adoption of a smaller threshold for the t-statistics, e.g., *t* = 3 as in Betti et al. (2018), produced very similar results. We are aware that the choice of this threshold certainly influences the size of the obtained components and thus to draw any conclusions on its absolute value within a condition can be misleading, also due to the contribution of false positive links in a component (Zalesky et al., 2012). Nevertheless, in this work we adopted the same threshold in all bands when comparing GCS-corrected vs. non-corrected data. For this reason, since all the data will be affected in the same way by the choice of the threshold, we limited our observations on the relative increase or decrease of the component size observed across frequency bands.

The significance level of each component was set to *a* < 0.01 assessed through permutation testing (number of permutations *n* = 200). Then, we adopted the MATLAB toolbox BrainNet Viewer for the graph visualization (Xia et al., 2013).

### Graph Analyses

In this work, the graph measures were computed on the BLP connectomes averaged across subjects. Thus, from the z-score connectivity matrices estimated for each run, obtained after Fisher transformation of the original correlation values, we first averaged across runs and then across subjects to obtain mean connectivity matrices. This applies both for the GCS corrected and the uncorrected data. Then, we thresholded the computed matrices to obtain fully connected binarized connectomes (Bordier et al., 2017). To this aim, the graph components were estimated at several thresholds. Then, the maximum threshold ensuring a single graph component, and thus full connectedness, was selected. This procedure allowed us to compare graphs in the same condition (a path between any pair of nodes exists), across uncorrected and leakage corrected scenarios and across bands. Now, to characterize the topology of the graph, measures such as centrality, connection density, modularity and efficiency were computed on these binarized connectomes. To quantify the centrality of the graph nodes, we adopted the binary Betweenness Centrality (BC) that is related to the number of times a node acts as a bridge between the strongest connections of any two nodes. Thus, nodes with high BC participate in many shortest paths. The BC is computed according to the following formula:

$$B\,C\,\left(v\right)=\frac{2}{\left(N-1\right)\,\left(N-2\right)}\,\sum_{i\neq j\neq v}\frac{\sigma_{i\,j}\,\left(v\right)}{\sigma_{i\,j}}$$

where σ_*ij*_ is the total number of shortest paths from node *i* to node *j*, σ_*ij*_(*v*) is the fraction of those paths passing through the node *v* and *N* is the graph order (Rubinov and Sporns, 2010; Sporns, 2011). To compare the pattern of BC values across different conditions (GCS corrected and non-corrected graphs) and bands, this was normalized to the sum of BC across nodes.

Notably, to assess the significance of the BC, we compared the obtained values with those from a population of random graphs. These were generated by the approach described in Rubinov and Sporns (2011) where a randomization function preserving the degree and strength distributions was employed (each graph edge was randomly rewired five times and 1000 iterations were run). From the obtained population of random graphs, we computed the 95th percentile of the obtained distribution and we considered as significant only those BC values exceeding such value.

To characterize the global integration properties of the graph we adopted the Global Efficiency (GE), a measure related to the information exchange across the whole graph (Rubinov and Sporns, 2010; Sporns, 2011). GE is defined as the average inverse shortest path length in the network, which is inversely related to the characteristic path length:

$$G\,E=\frac{1}{N\,\left[N-1\right]}\,\sum_{i,j\in N,i\neq j}\frac{1}{d_{i\,j}}$$

where *d*_*ij*_ is the shortest path length between the nodes *i* and *j*. In order to evaluate significant effects of the GCS on GE, we performed a paired *t* -test comparing GE obtained with leakage correction and without it, across resting state sessions, within each frequency band. Probability values were Bonferroni corrected for multiple comparisons.

We further investigated how eventual changes in GE related to the graph Modularity and Density of connections. Modularity is a measure that identifies the community structure of a network as a subdivision of non-overlapping groups of nodes in a way that maximizes the number of within-group edges and minimizes the number of between-group edges. Thus, this represents a statistic related to the degree to which the network may be subdivided into clearly delineated modules. Different approaches can be adopted to this aim, here we used the Louvain algorithm which provides a fast and accurate community detection (Blondel et al., 2008). We also checked that any modulation of the GE was not a mere effect of a different density of connections. The effect of the GCS on these two measures was also assessed with paired *t* -tests, with significance Bonferroni-corrected for multiple comparisons.

All the above quantities have been estimated by means of the MATLAB toolbox, the Brain Connectivity Toolbox^1^ (Rubinov and Sporns, 2010) and for graph visualization the BrainNet Viewer^2^ (Xia et al., 2013) was adopted.

### Control Analyses Using Orthogonalization

In order to compare the GCS approach with another popular leakage correction approach, we performed the same topological analyses described in the previous subsections on orthogonalized connectomes. Signal orthogonalization is another correction scheme based on linear regression to remove all zero-lag correlations (Brookes et al., 2012; Hipp et al., 2012). A multivariate version designed for beamformer inverse solution, ensuring symmetrical connectomes has also become widespread (Colclough et al., 2015), but we did not apply it since spatial leakage is symmetrical in minimum norm estimates. Here, we used the basic pairwise linear regression of Brookes et al. (2012). Specifically, given a seed **r**_0_, the orthogonalized vector activity was obtained by regressing out the seed signal ***Ψ***_**r**0_(*t*):

$${\text{Ψ}}^{O\,R\,T}\,\left(t\right)=\text{Ψ}\,\left(t\right)-\text{β}\,{\text{Ψ}}_{{\text{r}}_{0}}\,\left(t\right),$$

where the 3N_voxels_×3 matrix $\text{β}={\text{ΨΨ}}_{r\,0}^{+}$ encodes the regression coefficients. In this last equation, ***Ψ*** gathers all samples ***Ψ***(*t*) of the activity vector in a 3N_voxels_ × N_time_ matrix, and correspondingly the seed activity ***Ψ***_**r**0_(*t*) is a 3 × N_time_ matrix.

As in the GCS case, the row of the connectivity matrix corresponding to each seed was obtained by correlation of the BLP obtained from the uncorrected activity ***Ψ***_**r**_0__(*t*)at the seed and the BLP of all the corrected activities ***Ψ***^*ORT*^(*t*)at the other nodes.

Orthogonalized connectomes were then symmetrized, to account for numerical errors. The distance mask over node pairs closer than 35 mm was applied also in this case, given that orthogonalization also leads to similar under-correction effects (Palva et al., 2018), albeit less extensively than the GCS (Wens et al., 2015).

As in the GCS vs. uncorrected case, we ran the following analyses: (i) we compared the mean z-Fisher correlation values between uncorrected and orthogonalized connectomes; (ii) we applied NBS at each frequency band, using a threshold for the t-statistics equal to 6, as for the GCS vs. uncorrected comparison; (iii) given the disruptive effect of orthogonalization on the *z* -score correlation values, we also used a higher t-threshold, namely 10, to display and discuss the component size; (iv) we binarized the band-specific, group-averaged, orthogonalized connectomes imposing the full connectedness, as for the uncorrected and GCS connectomes; (v) we estimated BC and its significance for each node in the graph; (vi) we estimated GE, graph modularity and density and we compared these values with the ones obtained from the uncorrected connectomes through a *t* -test, with significance Bonferroni-corrected for multiple comparisons.

## Results

### Global Topology of GCS vs. Uncorrected Connectomes

**FIGURE 1:**
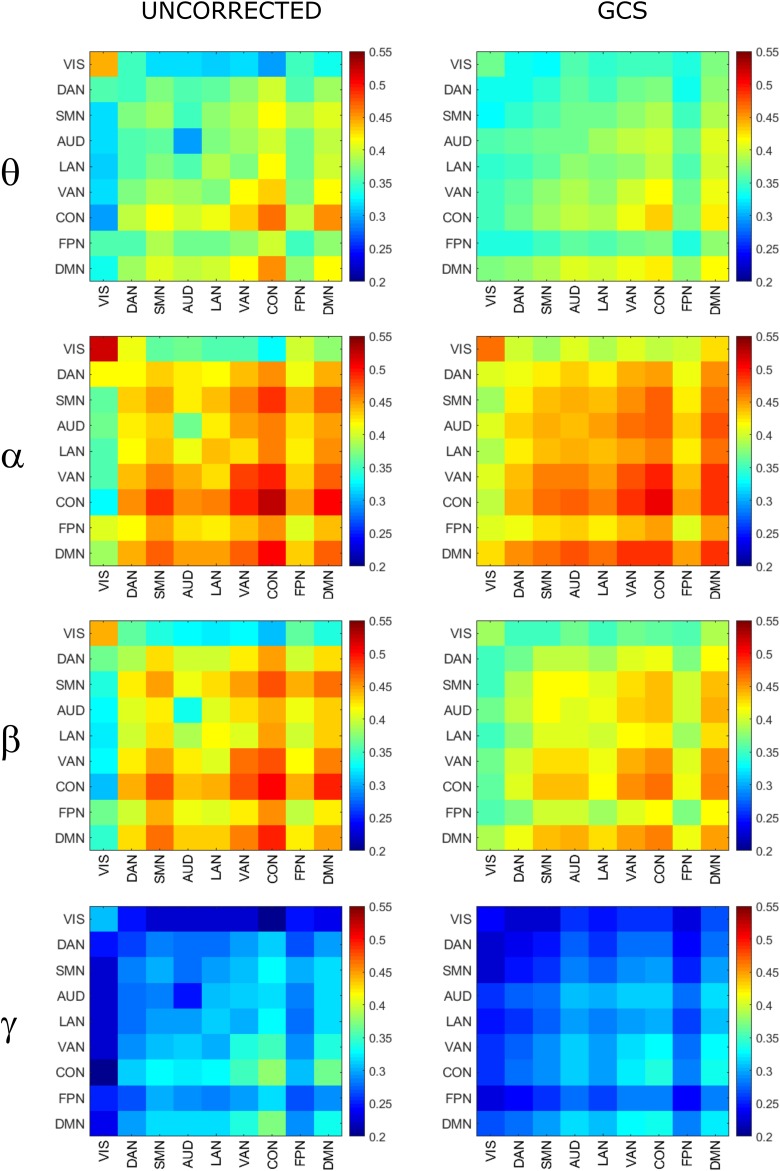
Effects of GCS on correlation strength. Network-based functional connectomes obtained as the average correlation over RSN nodes (see Table 1), are shown without (UNCORRECTED, **left column**) and after GCS **(right column)** leakage correction for each frequency band.

To investigate the effect of signal leakage correction on the architecture of within and across RSN interactions, we computed RSN-based connectomes, obtained by averaging the dense connectomes first across the RSN nodes (see Table 1 for a complete list of nodes, RSNs and abbreviations), discarding the node pairs closer than 35 mm, and then across subjects. The results for the uncorrected and GCS-corrected data are shown in Figure 1 (left column: UNCORRECTED, right column: GCS) at the different frequency bands. Although a spatial modulation of the RSN-based connectome is apparent across all bands, local reductions and increases of connectivity seem to be balanced. In fact, the connectivity averaged over the whole connectome does not differ significantly with and without GCS (*t* -tests over subjects, all *p* s > 0.35 for all bands). This suggests that GCS might preserve the global strength of connectivity. In addition, these RSN-based connectomes reveal a common modulation of connectivity patterns: within-VIS correlation seems to decrease for all bands after GCS, while within-AUD correlation seems to increase.

**TABLE 1:** List of abbreviations of the node and network labels adopted in this work.

	**MNI Coordinates**			Label	RSN		**MNI Coordinates**			Label	RSN
	**x**	**y**	**z**				**x**	**y**	**z**		
1	30.3	−12.8	52.6	rFEF	DAN	52	24	−34	60	RdPoCe	SMN
2	−26.3	−11.8	52.7	lFEF	DAN	53	−24	−32	63	LdPoCe	SMN
3	23.2	−69.4	48.6	rpips	DAN	54	−54	−23	37	LvPoCe	SMN
4	46	−32	50	RaIPs	DAN	55	54	−18	37	RvPoCe	SMN
5	18	−59	53	RdSPL	DAN	56	−24	−20	70	LdPrCe1	SMN
6	28	−49	52	RSPL	DAN	57	−44	−9	15	LmI	SMN
7	26	−69	30	RvIPS2	DAN	58	22	−18	58	RdPrCe	SMN
8	−25.3	−67.3	47.6	lpips	DAN	59	11	−46	59	RSPL−preCun	SMN
9	−32	−42	45	LaIPS	DAN	60	−18	−36	55	LdCS	SMN
10	−22	−53	52	LSPL	DAN	61	−7	−44	56	LdmSPL	SMN
11	−31	−80	18	LvIPS1	DAN	62	9	−43	53	RmdSPL	SMN
12	−43.4	−71.9	−7.7	lmt	DAN	63	10.68	−91.88	6.1	rV1	VIS
13	42.4	−69.7	−11.2	rmt	DAN	64	−2.82	−100.76	−0.49	lV1	VIS
14	53	−28	36	RSMG	DAN	65	−8	−88	−7	LV1v	VIS
15	45	−3	34	RvPrCe	DAN	66	14.29	−95.7	13.63	rV2	VIS
16	46	−51	−14	RvITG1	DAN	67	−7.26	−98.79	6.79	lV2	VIS
17	39	30	12	RIFG	DAN	68	27.5	−88.5	14.5	rV3	VIS
18	−49	−5	32	LvPrCe	DAN	69	−16.11	−92.7	17.83	lV3	VIS
19	−40	−42	−19	LvITG1	DAN	70	26.67	−71.44	−13.94	rV4	VIS
20	−45	−34	45	LPoCe	DAN	71	−30.98	−76.54	−17.13	lV4	VIS
21	−53	−29	37	LvPoCe	DAN	72	32.23	−78.38	25.12	rV7	VIS
22	41	17	31	RMFG	VAN	73	−23.1	−78.13	26.12	lv7	VIS
23	12	5	61	RMFG2	VAN	74	−7	−86	36	LV7−POSd	VIS
24	41	2	50	RMFG3	VAN	75	−22	−71	6	LV7	VIS
25	52	−48	28	RSMG	VAN	76	34	−88	−4	RLO	VIS
26	58	−48	10	rSTG	VAN	77	−40	−78	−9	LMT	VIS
27	40	21	−4	RIFG	VAN	78	−33	−86	10	LLO	VIS
28	46	11	9	RvIFG	VAN	79	37	−62	−11	RVOIT	VIS
29	27	50	23	RaPFC	VAN	80	43	−75	−11	RLOMT	VIS
30	−57	−48	32	LSMG	VAN	81	−25	−93	−2	LFovea	VIS
31	5	−22	40	RAC2	VAN	82	42	−80	4	RLOMT	VIS
32	30	8	−5	RAI	VAN	83	35	−79	0	RLO	VIS
33	−44	10	8	LvIFG	VAN	84	−11	−74	−6	LVP	VIS
34	42	28	1	RIFG−AI	VAN	85	17	−64	−5	RVP	VIS
35	−33	17	−5	LAI	VAN	86	18	−76	26	RPOSd	VIS
36	−10	−32	43	LPC	VAN	87	60	−22	6	rMSTG	AUD
37	39	11	21	RvPrCe	VAN	88	−41	−28	6	lMSTG	AUD
38	−60	−28	24	lSII	SMN	89	−51	−22	5	LmSTG	AUD
39	35	−26	55	rCS	SMN	90	−56	−33	16	LSTG1	AUD
40	56	−2	23	RPrCe	SMN	91	−43	−34	11	LSTG2	AUD
41	−37	−19	53	lCS	SMN	92	54	−43	12	rIST	AUD
42	−57	−8	21	LPoCe	SMN	93	−54	−40	10	lIST	AUD
43	57	−28	23	rSII	SMN	94	38	−19	12	RpI	AUD
44	−1	−17	55	lSMA	SMN	95	−35	−20	14	LpI	AUD
45	−10	−12	60	lSMA2	SMN	96	34	−24	17	RpI	AUD
46	−12	−20	40	lSMA3	SMN	97	36	0	12	RmI	AUD
47	4	−15	53	rSMA	SMN	98	−38	−4	11	LmI	AUD
48	10	−33	52	RParaCe	SMN	99	50	−12	17	RmI	AUD
49	−30	−18	10	lPUT	SMN	100	38	−6	4	RmI	AUD
50	30	−17	9	rPUT	SMN	101	−37	−8	3	LmI	AUD
51	−42	−17	46	LcPrCe	SMN	102	−30	0	15	LmI3	AUD
103	−48	31	−1	IFG	LAN	130	−19	22	52	LSFG	DMN
104	−50	19	9	lifg2	AUD	131	17	24	51	RSFG	DMN
105	−45	13	24	MFG	LAN	132	13	40	40	RSFG2	DMN
106	−7	9	60	lmfc4	AUD	133	59	−25	−12	RSTS	DMN
107	−50	−54	22	STS	LAN	134	−5	31	−4	LAC3	DMN
108	−56	−12	−3	aSTG	LAN	135	−9	36	8	LAC2	DMN
109	−55	−48	15	pSTG	LAN	136	−3	28	55	LmSFG2	DMN
110	−48	−44	3	lstg3	AUD	137	52	−1	−25	RMTG1	DMN
111	−56	−33	3	lstg2	AUD	138	−6	16	63	LmSFG1	DMN
112	−52	−54	12	lstg4	AUD	139	42	10	−29	RMTG2	DMN
113	−54	−23	−3	lstg1	AUD	140	−41	7	−31	LITG	DMN
114	47	25	−4	rifg1	AUD	141	−31	−59	42	LIPS	FPN
115	44	−36	6	rstg2	AUD	142	30	−61	39	RIPS	FPN
116	53	23	7	rifg2	AUD	143	51	−47	42	RIPL	FPN
117	61	−43	8	rstg1	AUD	144	10	−69	39	RprCu	FPN
118	2	52.6	23.5	rMPFC	DMN	145	−43	22	34	LdlPFC	FPN
119	−2	50.5	1.7	lMPFC	DMN	146	−9	−72	37	LprCu	FPN
120	−13.1	51.5	23.4	lMPFC2	DMN	147	43	22	34	RdlPFC	FPN
121	−2	40	27	LmPFC2	DMN	148	−41	3	36	LFC	FPN
122	−3	−54	31	lPCC	DMN	149	−51	−51	36	LIPL	FPN
123	51	−64	32	rAG	DMN	150	−28	51	15	LaPFC	FPN
124	−43	−76	35	lAG	DMN	151	39	1	42	RdPrCe	FPN
125	−56.6	−25.1	−16.9	lITG	DMN	152	−33	13	9	LaI_CO	CON
126	8	−51	29	RPCPreCun	DMN	153	36	16	4	RaIfO	CON
127	0	−65	31	RPreCun	DMN	154	−1	10	46	dACCmsFC	CON
128	−1	44	−2	LAC1	DMN	155	8	3	51	RpreSMA	CON
129	−4	42	45	LmSFG3	DMN						

**FIGURE 2:**
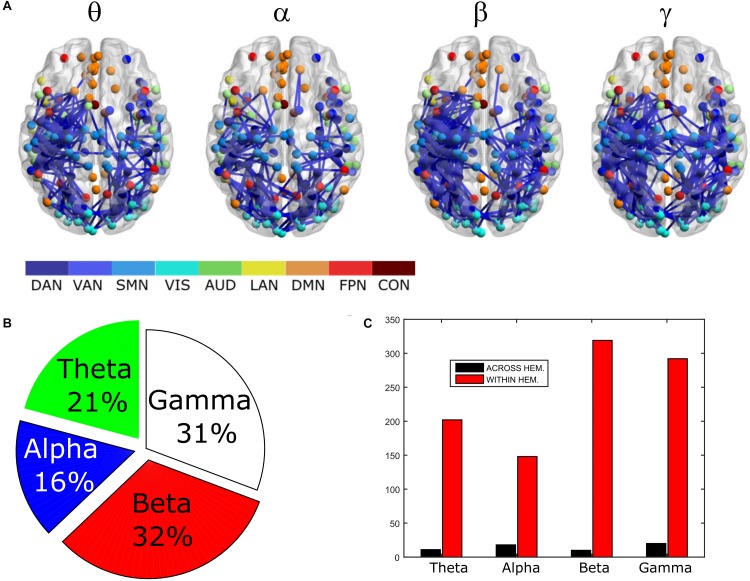
Analysis of global topology: decreased components after GCS. Results of the NBS on uncorrected and corrected connectomes are shown for each frequency band. **(A)** Decreased components (blue) displayed over an MNI brain. **(B)** Relative sizes of the decreased components (normalized to the total number of decreased links across bands). **(C)** Number of links, in the decreased components, changing within (red bars – WITHIN HEM.) and across hemispheres (black bars – ACROSS HEM.).

**FIGURE 3:**
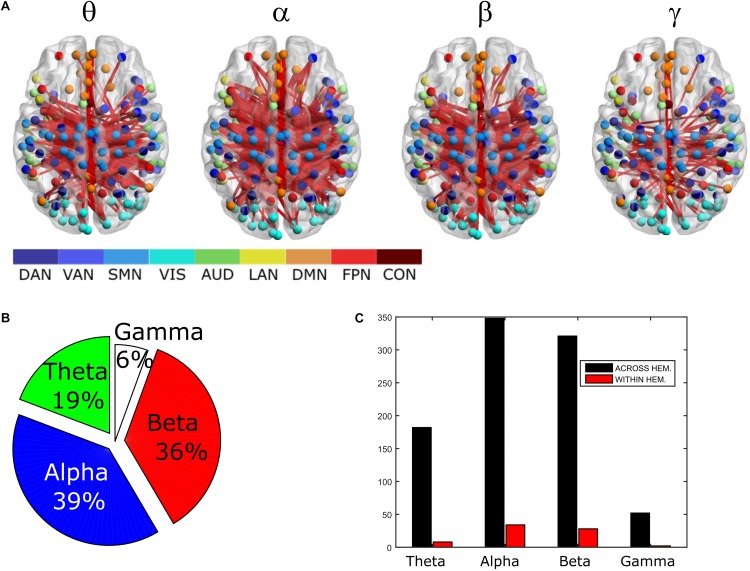
Analysis of global topology: increased components after GCS. Results of the NBS on uncorrected and corrected connectomes at each frequency band. **(A)** Increased components (red) displayed over an MNI brain. **(B)** Relative sizes of the increased components (normalized to the total number of increased links across bands). **(C)** Number of links, in the increased components, changing within (red bars – WITHIN HEM.) and across hemispheres (black bars – ACROSS HEM.).

In order to assess the statistical significance of the topological changes underlying these modulations, we applied NBS to the dense connectomes to identify graph components containing significantly different connections. As it can be seen in Figure 2A, when comparing uncorrected with GCS-corrected connectomes, we obtained components representing significant GCS-induced decrements of BLP correlation in all the physiological bands. These results are overlaid onto an MNI reference brain (BrainNetViewer, Xia et al., 2013) and nodes are color-coded based on the membership to fMRI RSNs (see reported colormap in Figure 2). Notably, at the same t-statistics threshold for all bands, the number of links in the decreased component assumed a different size as a function of the frequency band. Specifically, the decreased components in the beta, gamma and alpha bands involved a larger number of nodes than in the theta band. The pie chart reported in Figure 2B shows the percentage of links significantly decreased after GCS in each frequency band with respect to the total number of links decreased across all bands, which was 1022 at the t-threshold = 6. It can be noted a similar proportion of links removed in the beta/gamma bands (around 30%) followed by the theta (21%) and alpha band (only 16%). Interestingly, Figure 2C shows that the majority of links removed by GCS were intra-hemispheric (WITHIN HEM. – red bars), as compared to the inter-hemispheric ones (ACROSS HEM. – black bars). We acknowledge that care should be taken in interpreting the number of components’ nodes and edges for each band, since NBS controls the family wise error rate in the weak sense. Thus, the significance is associated to the whole component and not to single links, some of which might represent false positives (Zalesky et al., 2012). However, since we used the same t-test threshold across bands, we expect approximately the same number of false positives in all bands and thus the comparison of the number of edges in the decreased components in the beta and gamma bands versus the other bands is reasonable. In addition to components representing significant decreases, we also obtained components representing significant increases in all bands (see Figure 3A). In this case, the majority of increments in the number of links occurred in the alpha and beta bands and to a lesser extent in the theta band, while the gamma band involved fewer links. The percentage of links (with respect to the total number of increased connections across all bands, which was 1022) increasing after leakage correction was approximately 40% for alpha and beta bands and it decreased to about 20 and 6% in theta and gamma bands, respectively (see Figure 3B). Again, because of false positives, the absolute number of supra-threshold links should be interpreted cautiously. However, the main result is that the size of the increased component in the alpha and beta bands is considerably larger than for the theta and gamma bands. Furthermore, as it can be seen in Figure 3C, most involved links were between hemispheres (black – ACROSS HEM.) and only a few links were within hemispheres (red – WITHIN HEM.). To summarize, the GCS considerably changed the topology of the interactions, producing local, within-hemisphere decreases, and long-range, between-hemisphere increases. These effects were stronger in the beta and gamma bands for the decreases, and in the alpha and beta band for the increases.

### Integration/Segregation in GCS vs. Uncorrected Connectomes

**FIGURE 4:**
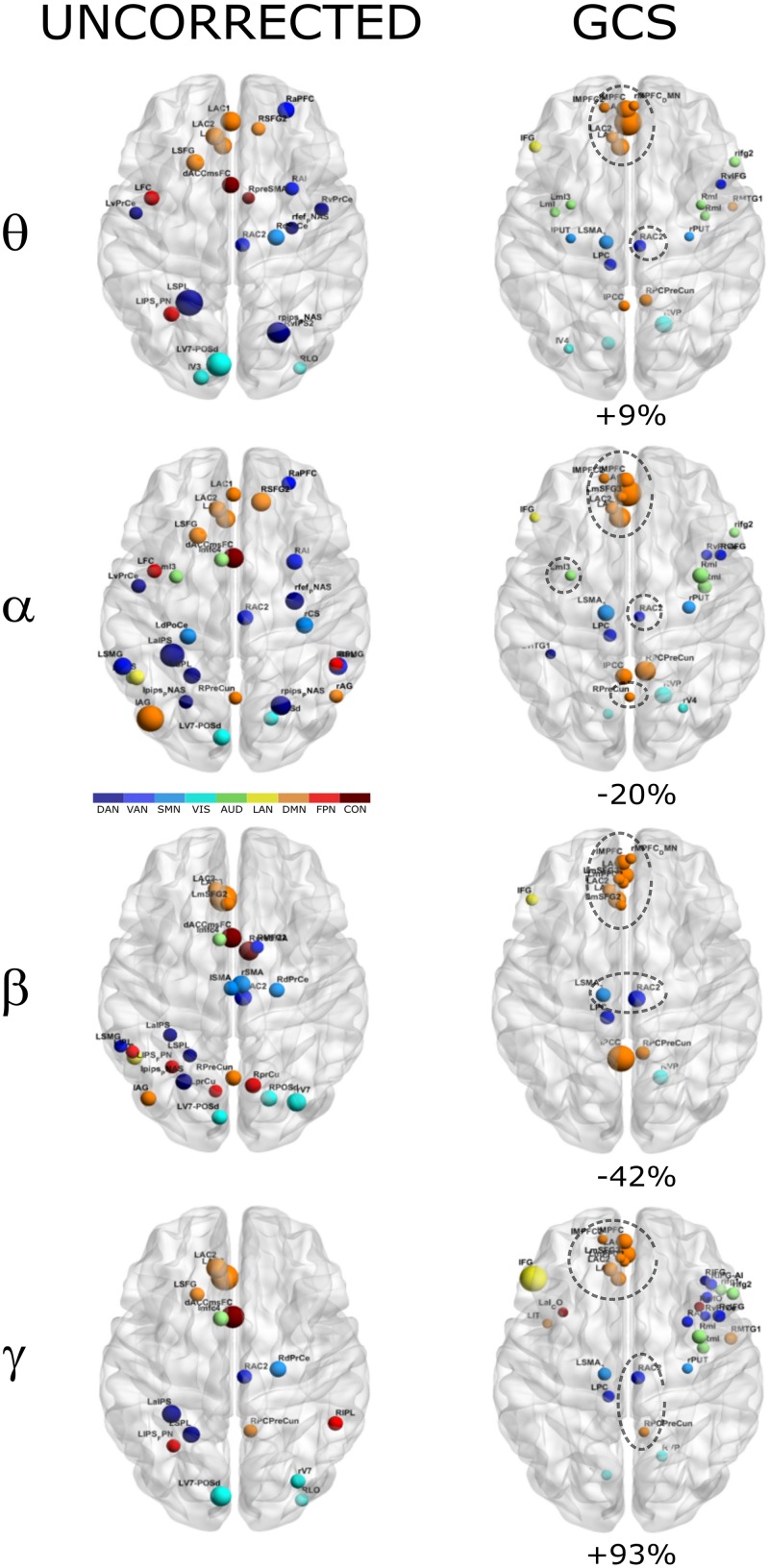
Functional hubs before and after GCS. Functional hubs identified through the BC are reported at the considered frequency bands without (UNCORRECTED) and with (GCS) leakage correction. A set of hubs belonging to the DMN (orange) and SMN (light blue) are consistently observed (dotted circle) after the leakage correction. For each frequency band, the percentage increase of the number of hubs due to the GCS is reported.

In order to investigate how the modulation of topology affected the integration and segregation in the whole brain network, we computed the Betweenness Centrality of the considered nodes, over the binary graphs obtained from uncorrected and GCS-corrected connectomes, in each frequency band (see Figure 4). First, we note that leakage correction led to a difference in the number of significant hubs: we obtained a slightly larger number of hubs in the theta band (+9%) as compared to the uncorrected data, while in the gamma band the number of hubs almost doubled (+93%). On the contrary, in the alpha (−20%) and beta (−42%) bands the GCS reduced the number of central regions. This presumably indicates that in these bands the centrality of uncorrected graphs was inflated by local spurious connections due to spatial leakage. Interestingly, apart from the observed changes, it must also be noted that some hub regions remained central both with and without GCS (see dashed contours in Figure 4). In all bands, medial prefrontal and posterior cingulate/precuneus nodes (except for the theta band) of the DMN remained central after leakage correction. Similarly, we found consistent hubs near/at the Supplementary Motor Area part of the SMN. In contrast, the IPS region, part of the DAN, did not result central after GCS. In summary, the topology of central nodes is partially maintained after the GCS.

**FIGURE 5:**
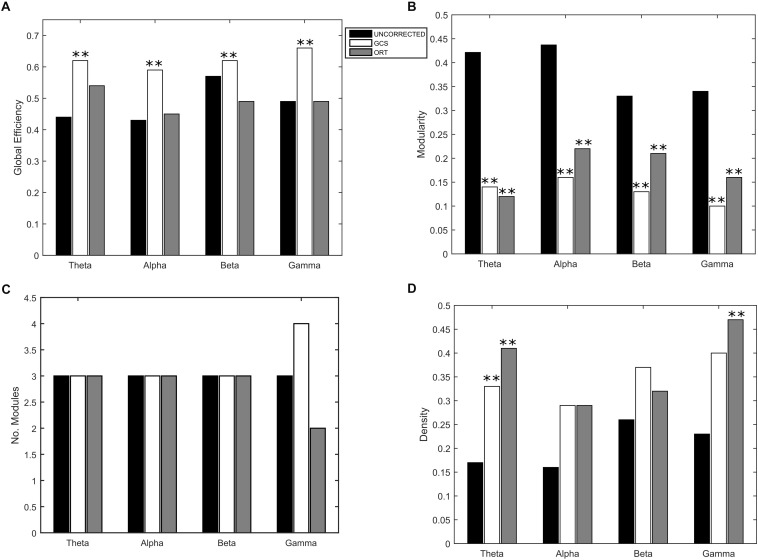
Effect of GCS and ORT on the global integration. **(A)** Mean Global Efficiency (GE) values computed before (black bars – UNCORRECTED) and after leakage correction (GCS- white bars, ORT – gray bars). A statistically significant difference was found in each frequency band (^∗∗^*p* < 0.01, Bonferroni corrected) for the GCS corrected data. The GCS seems to increase the efficiency of communication in the brain. This does not apply to the ORT correction where no statistically significant difference was found. **(B)** Mean modularity as a function of the frequency band. All the differences were statistically significant both for GCS and ORT (^∗∗^*p* < 0.01). The decrease of modularity shows that, after GCS and ORT, the segregation of the functional communities is lower. **(C)** The average number of modules is constant in all frequency bands except for gamma, despite the t-statistics is not significant. **(D)** No significant increase of the average density of connections is observed in general, apart from the theta band for GCS and theta and gamma for ORT (^∗∗^*p* < 0.01).

Eventually, to understand the effect of the GCS on the global integration, we estimated the GE of communication, which measures the overall efficiency of integration of the considered graph. As it can be seen in Figure 5A, we obtained a statistically significant (*p* < 0.01, Bonferroni corrected) increase of GE in GCS (white bars) vs. uncorrected (black bars) connectomes across all frequency bands. This suggests that the GCS might lead, in general, to a more efficiently integrated connectome as compared to the uncorrected data. Then, since we observed a loss of local links and an increase of long-range ones, to interpret the GE modulation we addressed some further graph measures that might influence the GE. First, we computed the Modularity (Figure 5B) and the number of modules (Figure 5C) that can be identified in the average connectome. As it can be seen in Figure 5B, a general decrease of modularity due to GCS was obtained in all bands (*p* < 0.01, Bonferrroni corrected), while the number of modules did not significantly change in all bands (Figure 5C). These results, taken together, are fundamental since they show that while the number of functional communities remain the same (same number of modules), they are less segregated once leakage corrected (smaller modularity). As a fundamental control on the GE modulation, we considered the density of connections in every band (Figure 5D). This is necessary because GE and density are known to be dependent, i.e., higher density leads to higher GE, and at least in a certain

regime such dependence is linear (Fornito et al., 2016; Strang et al., 2018). Thus, one may suspect that the higher values of GE obtained with GCS are simply driven by higher density. To address this issue, we first statistically tested the densities obtained at the different frequency bands, see Figure 5D. As it can be noted, although on average the density values in GCS are larger than in uncorrected connectomes, a paired *t* -test showed that these are not significantly different (apart from the theta band, *p* < 0.01, Bonferroni corrected). This provides a first indication that the GE differences might not be ascribed to the density variations. However, since the obtained density values lie in the range where a linear relationship is expected between GE and density (see Strang et al., 2018), we performed a regression analysis to check whether the density may predict the GE differences. We obtained no significant effects in the alpha and beta bands (*p* < 0.01, Bonferroni corrected). These results suggest that in these bands, the influence of density changes have a minor impact on the observed increase of integration for the GCS.

### Global Topology and Integration/Segregation in Orthogonalized vs. Uncorrected Connectomes

**FIGURE 6:**
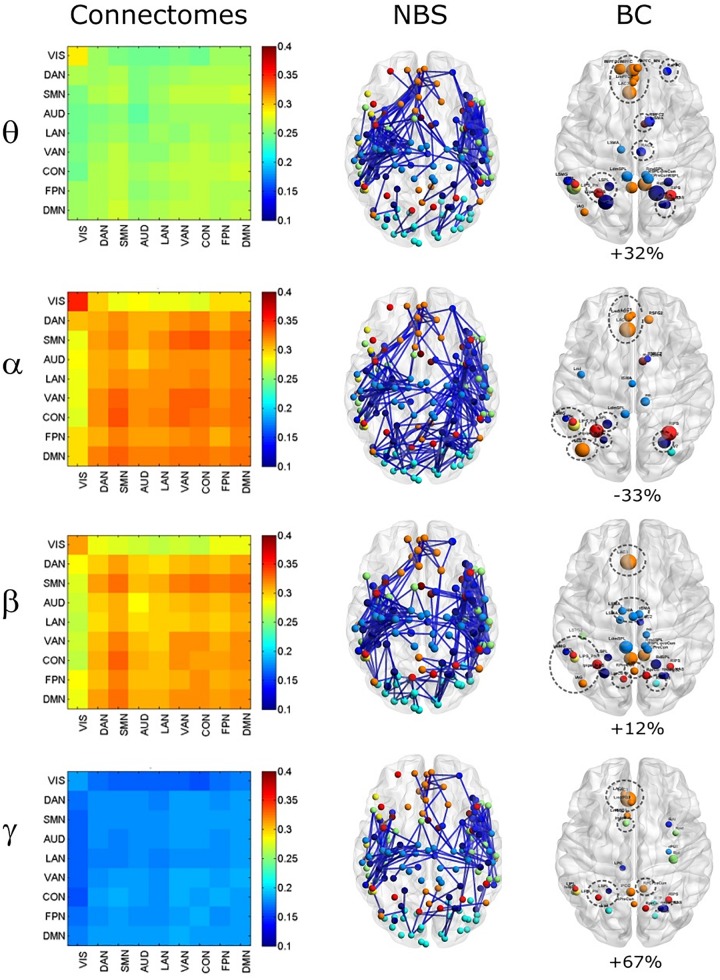
Effects of ORT on Correlation strength, global topology and centrality. **Left column:** Network-based functional connectomes obtained as the average correlation over RSN nodes (see Table 1) and runs, are shown after the ORT leakage correction for each frequency band. **Middle column:** NBS decreased components (blue), displayed over an MNI brain, after the ORT correction. **Left column:** Functional hubs identified through the BC after ORT. The percentage increases of the number of hubs after ORT is reported and the dotted circles represent hubs in common with the uncorrected data.

To compare the impact of the GCS approach with current and popular leakage correction schemes, we performed the same analyses on data corrected through the orthogonalization approach described in the Methods section. The orthogonalized RSN-based connectomes are shown in Figure 6, left column. Please note that a scale different from Figure 1 was adopted to appreciate the connectome patterns (connectomes with the same scale of the uncorrected ones are reported in Supplementary Figure S1, left column). Further, to be consistent with the previous comparisons, correlations of node pairs closer than 35 mm were not considered in these analyses. It can be noted that, differently from the GCS, in this case, the correlation averaged over the whole connectomes considerably decreased in all bands (*p* < 0.0002, Bonferroni corrected). However, when inspecting Figure 6, some patterns in the uncorrected and orthogonalized RSN-based connectomes seem to be preserved: within-VIS correlation seems to be stronger than VIS-other RSNs in all bands, and the relative difference between within-AUD correlation and the averaged correlation seems to be positive, as with the GCS. Accordingly, when comparing orthogonalized and uncorrected dense connectomes, NBS produced only components representing significant decreases (see Figure 6, middle column and Supplementary Figure S1, right column). If the same t-statistics threshold as for the uncorrected vs. GCS comparison is applied (Supplementary Figure S1), the components sizes are large for all bands and involve all the nodes of the connectomes. Specifically, the percentage of edges (with respect to the total number of decreased edges across all bands) in the decreased components is similarly shared across bands (about 25% in theta, 28% in alpha, 26% in beta, and 21% in gamma). For display and interpretation purposes, we show the results of NBS statistics using a higher threshold (*t* = 10, middle column in Figure 6) to appreciate possible differences across bands. In this case, the decreased components in alpha and beta bands involved a larger number of nodes (about 130) than in theta and gamma bands (about 100). The percentage of links with respect to the total number of decreased links across all bands reduced by orthogonalization is larger in alpha/beta bands (28%) followed by the theta (22%) and gamma band (only 16%). Analogously to the GCS, the majority of links removed by orthogonalization were intra-hemispheric (209 in theta, 243 in alpha, 265 in beta, 101 in gamma), as compared to the inter-hemispheric ones (21 in theta, 33 in alpha, 22 in beta, 58 in gamma).

Moreover, the BC analysis on orthogonalized connectomes (see Figure 6, right column) revealed an increase in the number of hubs with respect to the uncorrected connectomes in the theta and gamma bands, and a decrease in the alpha band, as occurred for GCS-based connectomes (+32%, +67%, and −33% respectively). However, differently from GCS, the number of hubs slightly increased in the beta band (+12%). Hubs in the medial prefrontal (in all bands) and posterior cingulate/precuneus nodes (in the beta and gamma bands) of the DMN, together with hubs near/at the Supplementary Motor Area part of the SMN (in the beta band) remained central, as with the GCS (Figure 6, contoured nodes, left column and Figure 4, left column). In addition, hubs in the IPS regions of the DAN and FPN (in all bands), and in the AG (DMN, alpha and beta bands), were found in both the uncorrected and orthogonalized connectomes. These results suggest that for orthogonalized matrices, a partial overlap with uncorrected data on the hub topography can be revealed. Interestingly, some of these hubs overlap also with those obtained after applying the GCS.

As far as it regards the integration analyses, the effects of orthogonalization were slightly different from the ones obtained for GCS (see Figure 5). Interestingly, in the orthogonalized data (ORT) no statistically significant increases in the GE were observed (Figure 5A, gray bars). Nevertheless, a significant decrease of modularity was observed across all bands (*p* < 0.01 Bonferroni corrected, Figure 5B), while the number of modules did not change, as with GCS (Figure 5C). The density of connections, although on average larger than in the uncorrected connectomes, reached statistical significance only in the theta and gamma bands (*p* < 0.01, Bonferroni corrected). In summary, the orthogonalization seems not to affect the global integration as measured via GE.

## Discussion

In this work, we analyzed the impact of the GCS in minimizing the effect of MEG spatial leakage on the topology of connectivity at rest. We compared the effect of GCS with a popular orthogonalization approach (Brookes et al., 2012). We focused on integration/segregation measures observing several interesting spatially- and frequency-specific aspects. First, we observed that the GCS significantly modified the overall topology: the connectivity decreased within each hemisphere, mainly in the gamma and beta bands, and increased across hemispheres, especially in the alpha and beta bands. On the other hand, orthogonalization only produced a decreased connectivity in every band, mainly involving intra-hemispheric links, as for GCS.

Second, in terms of BC, a set of hubs in the medial frontal and parietal areas, especially in the DMN and SMN, survived both leakage corrections, while more lateral regions (IPS) were preserved by orthogonalization but not by the GCS. Third, after GCS, we observed an increase of GE across all bands. This change occurred with a significant decrease in the Modularity corresponding to a constant number of modules, hence supporting a stronger integration among functional communities. The modulations of GE and Modularity, after GCS, could not be explained by a significant decrease in connection density (apart from the theta band).

### Impact of the Geometric Correction Scheme on the Overall Connectivity

The analysis performed by means of NBS on the overall connectivity structure showed that, despite the mean strength of GCS-corrected correlation was not significantly modified by leakage correction, a profound alteration of the topology was observed in all bands (Figures 2, 3), suggesting that uncorrected connectomes should be interpreted with care. In general, the first topological change consisted of a massive decrease of within-hemisphere connections among neighboring nodes (see Figure 2). This local effect is not surprising, since the leakage effect mainly consists of the influence of one source on its neighbors, due to the spatial spread of the reconstructed sources (Hauk et al., 2011; Wens, 2015; Wens et al., 2015) and their mis-localization. The edges involved in the decreased component were spatially located mainly in the parieto-occipital regions. Although leakage effects have different spatial distribution according to the location of the seed in the brain, the larger involvement of the parieto-occipital regions might simply reflect the higher density of the parcellation scheme in those areas. We also found that the size of the decreased components was spectrally specific, with larger components in the beta and gamma bands (Figure 2). It must be noted that although the spatial leakage itself is not frequency-dependent (Brookes et al., 2014; Wens et al., 2015) —a property that is built in the GCS— the induced effect on connectivity does depend on the frequency-specific signal-to-noise ratio (SNR) (O’Neill et al., 2015). Lower SNRs such as those expected in the theta and gamma bands tend to induce sharper local spurious connections. This may explain the larger component sizes found in beta, gamma, and theta as compared to alpha (which exhibits the largest SNR in MEG). Another possible explanation for the stronger post-GCS decreases in the beta and gamma bands could be a mitigation of the seed mis-location which affects the GCS (Wens et al., 2015). Specifically, seed mis-location, i.e., the position of the actual seed does not overlap with a true seed, generates spurious connectivity corresponding to a smaller loss of edges after GCS. Given that local synchronization at high frequencies is stronger, as reported in the influential work of Buzsaki and Draguhn (2004), the effect of mis-location is mitigated as there are many interacting seeds very close to each other.

Correspondingly, orthogonalization also led to massive connectivity decreases mainly involving intra-hemispheric links (Figure 6). However, this correction affected more links in the alpha/beta band, which exhibit high SNR, then in the theta and gamma bands, where SNR is lower. This might reflect an effect of SNR in the estimation of the regression coefficients, which could be biased by physiological bands with the larger power (i.e., alpha and beta). As such, orthogonalization tends to produce milder effects when the SNR is low. Alternatively, this difference could be ascribed to the existence of 0-lag interactions, which are preserved by the GCS but cancelled by orthogonalization. This is in line with the dominance of alpha/beta bands regarding connectivity increases post-GCS, that we discuss in the following.

The GCS yielded an increased component involving mainly edges connecting parietal nodes between hemispheres and edges connecting occipital and frontal nodes (Figure 3). An increase of connectivity was rather unexpected as leakage correction generically leads to lower connectivity due to the elimination of spurious couplings. However, some increased interhemispheric connectivity after leakage correction has already been reported when using orthogonalization or closely-related regressions (Brookes et al., 2012, 2014; Hipp et al., 2012; Maldjian et al., 2014; Wens et al., 2015), but they are milder and may be explained by an over-correction effect (Wens, 2015). Accordingly, in our data no such increases were detected with orthogonalization. Here, we report a global, not network-specific, increase of interhemispheric connectivity after GCS, suggesting that spatial leakage might screen some genuine, sufficiently long-range connectivity. Since a major distinction between the GCS and orthogonalization is the preservation of 0-lag interactions, this seems to suggest that this rather counter-intuitive effect relates to inter-hemispheric, short-lag correlations. In fact, we demonstrate mathematically in the Appendix that such screening emerges in the presence of linear correlation, which enables a non-linear contribution of spatial leakage to BLP correlations (despite its linearity in source activity). When the spatial leakage is strong, i.e., for local connections, it induces a spurious increase of short-range connectivity. This is in line with the results showing that both leakage corrections clean local connections mainly (Figures 2, 6). When spatial leakage is weaker, i.e., for longer-range connectivity, it may lead, through non-linear effects induced by 0-lag correlation, to a spurious reduction of connectivity. This fits with our results in Figure 3. As a matter of fact, in the uncorrected connectomes, the local connections composing the decreased components shown in Figure 2 were always significantly stronger than the long-range connections comprised in the increased components in Figure 3 (separate *t* -tests for each band, *p* < 10^–5^ for all bands).

Spectrally, as mentioned above, we noted a larger size of the increased component mainly in the alpha and beta bands which have been reported as the most consistent spectral signatures of fMRI RSNs (however, see Liu et al. (2017) showing that only whole band EEG RSNs match the spatial patterns of fMRI RSNs). These systems in fact cover mainly occipital, parietal and temporal regions and involve long-range interhemispheric or antero-posterior connections (Mantini et al., 2007; de Pasquale et al., 2010, 2012; Brookes et al., 2011; Hipp et al., 2012). Specifically, the alpha band was associated to the VIS, DAN, DMN, SMN, AUD in an EEG-fMRI study (Mantini et al., 2007); to SMN and DAN in an ECoG study (Hacker et al., 2017); and to VIS, VAN, LAN, SMN, DMN in MEG studies (Brookes et al., 2011, 2014; de Pasquale et al., 2012). Analogously, the beta characterized the CON in Mantini et al. (2007); the SMN, DMN, DAN, VIS, VAN, LAN, FPN in MEG studies (Brookes et al., 2011, 2014; de Pasquale et al., 2012). Eventually, a spatial concordance of these RSNs over a whole larger band was found also in Hipp and Siegel (2015). Interestingly, an increase of interhemispheric interactions in the alpha band in the VIS and in the beta band in the SMN was also observed after symmetrical orthogonalization in Colclough et al. (2015). In this work, we used a quite different pipeline (different inverse solver, keeping all components of source activity, denser connectome but based on pointwise source estimates, and the GCS for leakage correction) and extended Colclough’s findings identifying more extensive increases. This is possibly also in line with our mathematical description of the connectivity screening effect: symmetrical orthogonalization does not enforce strict pairwise orthogonalization but is rather based on a multivariate optimization, so some zero-lag correlations may survive and lead to connectivity increases post-correction as per our Appendix.

### Functional Hubs

In this work, the comparison between hubs identified with and without the leakage correction was based on the BC measure and the constraint of full connectedness of the investigated connectomes. We obtained that a set of regions survived the correction while others were ‘canceled,’ suggesting that they probably were spurious byproducts of spatial leakage. On the other hand, other regions resulted central after the correction (see Figures 4, 6). In fact, if the number of hubs slightly increased in the theta band and almost doubled in the gamma band, they decreased in the alpha. In the beta band, the number of hubs decreased with GCS and slightly increased with orthogonalization. Hubs that did not replicate with GCS were those in the IPS region, part of the DAN; while new hub regions were identified in right prefrontal cortex part of the Auditory and Ventral Attention Networks in the theta and alpha bands. As it can be seen in Figure 4, a set of interesting nodes survived the GCS and these areas comprised nodes from the DMN (orange) and SMN (blue). Apart from the specific nodes involved, the frontal areas of the DMN were consistent across frequency bands while the parietal nodes were mainly observed in the alpha and beta bands with some involvement of the gamma band. The preservation of hubs in the DMN and SMN in all bands was confirmed also after orthogonalization (Figure 6), which also maintained AG in the DMN and parietal nodes in the DAN and FPN, and introduced new hubs in the SPL-PreCun region.

The consistency of hubs observed across GCS and orthogonalization approaches nicely fit with previous MEG findings observed in de Pasquale et al. (2010, 2016, 2017, 2018) as well as fMRI findings (de Pasquale et al., 2013, 2017). Notably, at least for the parietal nodes of the DMN and SMN, our results extend the findings of Marino et al. (2019), in that the DMN hubs are mainly found from theta to beta bands, but at a lesser extent in the gamma bands, while the SMN hubs are also found in the gamma band, reflecting a balance between low- and high-frequency oscillations in the Cognitive (as the DMN) and the Perceptual (as the SMN) networks. The parietal regions of the DMN and, specifically, the Posterior Cingulate cortex/Precuneus have been shown to play a fundamental role of integration across several RSNs in the alpha and beta bands. Notably, these observations are in line with the work of Maldjian et al. (2014) where, in the leakage corrected MEG data, the DMN topography closely resembled the fMRI one and functional hubs were consistently observed in the posterior cingulate and bilateral parietal areas of this network. These results are interesting since they suggest that the centrality of these regions was not dominated by the leakage effects.

Analogously, SMN nodes like RAC2 (all bands for GCS, theta and beta for orthogonalization) and SMA (mainly in beta band for GCS and theta, alpha and beta for orthogonalization) were consistently observed both with and without leakage correction. These nodes have been previously reported as functional hubs of the Somato-Motor Network but more importantly these nodes together with DMN nodes seem to form a fundamental axis of integration related to the interplay between the internal vs external cognition (see for example de Pasquale et al., 2013) and lately part of a dynamic core network of integration (de Pasquale et al., 2016).

Finally, it is not unexpected that some differences between hub topographies was found when comparing the two leakage correction approaches. These differences could be partially ascribed to the contribution of the interhemispheric edges that increase with GCS but are not modified by orthogonalization. However, since the ground truth is unknown, fully proving this claim would require turning to simulated data and inspect the impact of the leakage correction methods on the estimated connectomes. This would require developing large-scale simulations of the brain connectome wherein topological features such as BC (and GE, as discussed below) are controlled, to compare estimated connectomes with known ground truth regarding network topology. Instead, we used here orthogonalization as a control to contrast with the GCS. In fact, Wens et al. (2015) presented small-scale but controlled network simulations that revealed two major differences: (i) orthogonalization suppresses linear, 0-lag correlations while GCS preserves it, and (ii) orthogonalization is more resilient than the GCS to local correction errors due to seed mis-location. Further, the simulations in Wens et al. (2015) showed that these local correction errors are confined to node pairs closer than 3 cm. Our use of a 35 mm mask thus mitigated this difference. In this setup, it is thus reasonable to expect that the preservation/cancelation of 0-lag correlations represent the only distinction between the two correction methods. As suggested by our mathematical model of the leakage “screening” effect (see Appendix), we expect our detection of post-GCS connectivity increases to be a consequence of that distinction. That said, full proof of this statement would require the above-mentioned large scale simulations of connectome topology, which thus represents an interesting avenue for future work. Until then, we suggest that both leakage corrections should be used to provide a more comprehensive overview on MEG functional architecture.

### Global Efficiency

The strong interaction realized by the functional axis DMN-SMN through the involvement of the hubs in the cingulate cortex has been shown to relate to a global optimization criterion (de Pasquale et al., 2016). Such topology seems to optimize the efficiency of information transfer as measured via the global efficiency. Now, since we confirmed the presence of these hub regions with leakage correction, it would be interesting to see their impact on these global measures. In fact, the effect of reducing connections (Figures 2, 6) might induce a decrease of the global integration. As it can be seen in Figure 5A this does not seem to be case: we observed a significant increase of GE in the alpha and beta bands after GCS (but not after orthogonalization). These results suggest that the spurious connections removed by GCS and orthogonalization from the connectomes were not serving the mentioned optimal criterion. On the other hand, the increase of interhemispheric links following GCS seems to promote the integration across the brain components. This result must be interpreted also considering the decrease of Modularity (Figure 5B) and the fact that the number of modules remained constant (see Figure 5C). Thus, in the leakage-corrected connectomes, it is more difficult to identify segregated communities (higher modularity) but not because the number of modules increased leading to more fragmented communities. In fact, the number of modules is constant, thus the same communities are, without the spurious contributions of spatial leakage, less segregated and more integrated, in the case of GCS. More generally, the topological effect of spatial leakage to spuriously increase of local connectivity explains its negative impact on optimal integration and modularity of the brain. Interestingly, we show in the Appendix that using orthogonalization, instead of the GCS, might tend to mitigate the detection of higher integration. This is confirmed by our results reported in Figure 5A where no significant changes in the GE were observed after orthogonalization.

## Conclusion

We showed that a proper leakage correction is necessary to study MEG functional topology and reinforces the findings that the brain functioning at rest relies on a topologically optimal local and global integration principle.

## Data Availability Statement

The datasets generated for this study are available on request to the corresponding author.

## Ethics Statement

This study was carried out in accordance with the recommendations of “Code of Ethics of the World Medical Association, and the Institutional Review Board and Ethics Committee at the University of Chieti” with written informed consent from all subjects. All subjects gave written informed consent in accordance with the Declaration of Helsinki. The protocol was approved by the Ethics Committee at the University of Chieti.

## Author Contributions

SD, MC, VW, and FP designed the research, interpreted the results, and wrote the manuscript. SD and FP performed the research and analyzed the data. SD, VW, and FP contributed analytic tools.

## Conflict of Interest

The authors declare that the research was conducted in the absence of any commercial or financial relationships that could be construed as a potential conflict of interest.

## Footnotes

1 https://sites.google.com/site/bctnet/

2 http://www.nitrc.org/projects/bnv/

## Supplementary Material

FIGURE S1 Effects of ORT on correlation strength **(left column)** with the same scale as Figure 1 and on global topology **(right column)** with the same t-threshold as Figure 2A.

## Appendix

### Analytical Demonstration of Connectivity Increases After GCS

To better understand the *a priori* counter-intuitive detection of higher connectivity values after GCS, we analyze here mathematically the effect of spatial leakage and characterize the changes brought by its correction. We demonstrate that: (i) the GCS decreases connectivity when leakage is strong enough (as expected for nearby sources), (ii) the GCS may increase connectivity for sufficiently weak leakage (as expected for well separated sources), and (iii) this increase occurs only in the presence of linear correlations. The last claim explains why no such connectivity increase was obtained after orthogonalization.

We consider source activity ***Ψ***_0_ at a seed location and ***Ψ***_1_ at a target location, which for simplicity will be assumed one-dimensional time courses. The linear mixing expected from linear, zero-lag spatial leakage can be written as

$${\text{Ψ}}_{1}={\text{Ψ}}_{1}^{G\,C\,S}+k\,{\text{Ψ}}_{0},$$

where ${\text{Ψ}}_{1}^{\text{GCS}}$ is the corrected target source activity and *k* denotes the coupling constant encoding the spatial leakage effect (Wens, 2015). As described in the Methods, the corresponding BLP time series are obtained by averaging the squared signal over windows *w*, so *B**L**P*_0,1_(*w*) = ⟨(***Ψ***_0,1_)^2^⟩_*w*_ and $B\,L\,P_{1}^{G\,C\,S}\,\left(w\right)={\left\langle {\left({\text{Ψ}}_{1}^{G\,C\,S}\right)}^{2}\right\rangle }_{w}$. The uncorrected BLP time course at the target source now depends quadratically on the leakage constant *k* :

$$B\,L\,P_{1}\,\left(w\right)=B\,L\,P_{1}^{G\,C\,S}\,\left(w\right)+k^{2}\,B\,L\,P_{0}\,\left(w\right)+2\,k\,L\,C\,\left(w\right),$$

where $L\,C\,\left(w\right)={\left\langle {\text{Ψ}}_{1}^{G\,C\,S}\,{\text{Ψ}}_{0}\right\rangle }_{w}$ is a time-dependent measure of linear coupling between the seed and corrected target sources. Finally, connectivity is computed using BLP correlation over the windows *w*. However, the root of the argument can be explained more easily by considering the BLP covariance:

$$c\,o\,v\,\left(B\,L\,P_{1},B\,L\,P_{0}\right)=c\,o\,v\,\left(B\,L\,P_{1}^{G\,C\,S},B\,L\,P_{0}\right)+k^{2}\,v\,a\,r\,\left(B\,L\,P_{0}\right)+2\,k\,c\,o\,v\,\left(L\,C,B\,L\,P_{0}\right).$$

Here the left-hand side represents uncorrected connectivity, and the first term in the right-hand side the corrected estimate. The second term is the coupling between the seed’s *BLP*_*0*_ and its linear contribution *k*^2^*B**L**P*_0_ to the target’s *BLP*_*1*_. Due to its positivity, it inflates the uncorrected connectivity, as may be intuitively expected. The third and key term in our argument depends on the covariation of the seed’s *BLP*_*0*_ and the time-dependent seed-target linear coupling *LC*. It can either increase or decrease the uncorrected connectivity, depending on the relative sign of *k* and *c**o**v*(*L**C*,*B**L**P*_0_). When this term is negative, it may or may not overcome the second, positive term depending on the leakage coupling strength |*k*|. The critical value *k*_c_ determined by the equality of these two contributions is

$$k_{c}=-2\,\frac{c\,o\,v\,\left(L\,C,B\,L\,P_{0}\right)}{v\,a\,r\,\left(B\,L\,P_{0}\right)}.$$

We conclude that: (i) When the leakage coupling is strong enough, i.e., |*k*| > |*k*_*c*_|, the second, positive term dominates, so $c\,o\,v\,\left(B\,L\,P_{1},B\,L\,P_{0}\right)>c\,o\,v\,\left(B\,L\,P_{1}^{G\,C\,S},B\,L\,P_{0}\right)$ and the GCS thus decreases connectivity. (ii) When the leakage coupling is weak, i.e., |*k*| < |*k*_*c*_|, the third term dominates, and if its sign is appropriate we have $c\,o\,v\,\left(B\,L\,P_{1},B\,L\,P_{0}\right)<c\,o\,v\,\left(B\,L\,P_{1}^{G\,C\,S},B\,L\,P_{0}\right)$ so the GCS may increase connectivity. (iii) Finally, this increase in connectivity requires the existence of a linear coupling *LC*, since otherwise *k*_c_ = 0 and the possibility (ii) cannot occur. Interestingly, orthogonalization strives to eliminate any linear, zero-lag correlation (in the most drastic case, non-stationary versions of orthogonalization set *L**C*(*w*)≈0 within each window, see O’Neill et al., 2015). So, using signal orthogonalization instead of the GCS would by design tend to mitigate this effect and thus may miss the increased optimization in brain integration reported in the main text.
