# Supplementary figures and images for: The Impact of the Geometric Correction Scheme on MEG Functional Topology at Rest
Source: Front Neurosci. 2019 Oct 25;13:1114. doi: 10.3389/fnins.2019.01114 (PMC6823434; doi:10.3389/fnins.2019.01114)

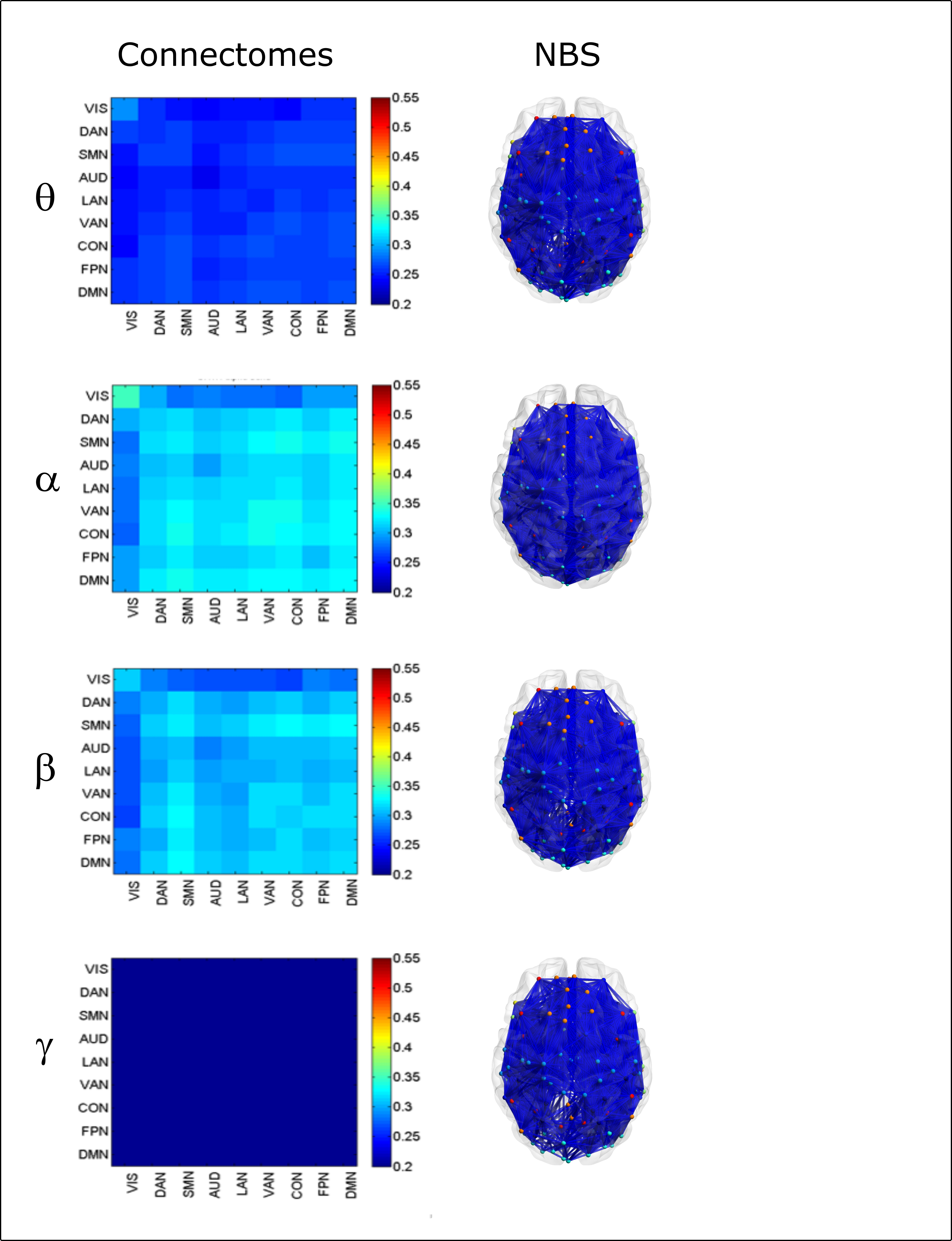

Supplement: FIGURE S1 — Effects of ORT on correlation strength (left column) with the same scale as Figure 1 and on global topology (right column) with the same t-threshold as Figure 2A. [file Image_1.TIF]
